# The expanding range of emerging tick-borne viruses in Eastern Europe and the Black Sea Region

**DOI:** 10.1038/s41598-023-46879-2

**Published:** 2023-11-14

**Authors:** Koray Ergunay, Brian P. Bourke, Drew D. Reinbold-Wasson, Mikeljon P. Nikolich, Suppaluck P. Nelson, Laura Caicedo-Quiroga, Nataliya Vaydayko, Giorgi Kirkitadze, Tamar Chunashvili, Lewis S. Long, Jason K. Blackburn, Nora G. Cleary, Cynthia L. Tucker, Yvonne-Marie Linton

**Affiliations:** 1 Walter Reed Biosystematics Unit, Museum Support Center MRC-534, Smithsonian Institution, 4210 Silver Hill Rd., Suitland, MD 20746-2863 USA; 2 One Health Branch, Walter Reed Army Institute of Research, 503 Robert Grant Ave., Silver Spring, MD 20910 USA; 3 Department of Entomology, Smithsonian Institution-National Museum of Natural History, 10th St NE and Constitution Ave NE, Washington, DC 20002 USA; 4 Department of Medical Microbiology, Virology Unit, Faculty of Medicine, Hacettepe University, Ankara, Turkey; 5 U.S. Army Medical Research Directorate-Georgia (USAMRD-G), Tbilisi, Georgia; 6 Bacterial Diseases Branch, Walter Reed Army Institute of Research, Silver Spring, MD 20910 USA; 7 Ukrainian Center of Diseases Control and Monitoring, Kyiv, 04071 Ukraine; 8 Uniformed Services, University of the Health Sciences, Bethesda, MD 20814 USA; 9 Emerging Pathogens Institute, University of Florida, Gainesville, FL 32610 USA; 10 One Health Center of Excellence, University of Florida, Gainesville, FL 32603 USA

**Keywords:** Metagenomics, Viral epidemiology, Viral evolution, Viral reservoirs

## Abstract

We analysed both pooled and individual tick samples collected from four countries in Eastern Europe and the Black Sea region, using metagenome-based nanopore sequencing (NS) and targeted amplification. Initially, 1337 ticks, belonging to 11 species, were screened in 217 pools. Viruses (21 taxa) and human pathogens were detected in 46.5% and 7.3%, respectively. Tick-borne viral pathogens comprised Tacheng Tick Virus 2 (TTV2, 5.9%), Jingmen Tick Virus (JMTV, 0.9%) and Tacheng Tick Virus 1 (TTV1, 0.4%). An association of tick species with individual virus taxa was observed, with the exception of TTV2, which was observed in both *Dermacentor* and *Haemaphysalis* species. Individual ticks from pools with pathogen detection were then further screened by targeted amplification and then NS, which provided extensive genome data and revealed probable pathogen Haseki Tick Virus (HTV, 10.2%). Two distinct TTV2 clades were observed in phylogenetic analysis, one of which included closely related *Dermacentor reticulatus* Uukuviruses. JMTV detection indicated integrated virus sequences. Overall, we observed an expansion of newly documented pathogenic tick-borne viruses into Europe, with TTV1 being identified on the continent for the first time. These viruses should be included in the diagnostic assessment of symptomatic cases associated with tick bites and vector surveillance efforts. NS is shown as a useful tool for monitoring tick-associated pathogens in pooled or individual samples.

## Introduction

The overall prevalence of tick-borne infections is increasing globally, due to the expansion of tick populations into new geographic regions and increased human exposure as a result of environmental and climatic changes^1,2^. Accounting for a major portion of vector-borne diseases in many countries, tick-borne infections may produce a significant disease burden on local healthcare providers and constitute an eminent public health threat^3^. With ticks acting as biological vectors, a wide palette of pathogenic microorganisms including viruses, protozoans and bacteria can be transmitted to humans and other susceptible vertebrates^3^.

Viruses transmitted by ticks are taxonomically heterogenous and exhibit diverse characteristics^4^. Those considered significant human or animal pathogens due to disease severity, distribution, potential to emerge and notable impact include Crimean-Congo hemorrhagic fever virus (CCHFV) and Nairobi sheep disease virus (NSDV) (family *Nairoviridae* genus *Orthonairovirus*); Tick-borne encephalitis virus (TBEV), Omsk hemorrhagic fever virus (OHFV), Kyasanur forest disease virus (KFDV), Powassan virus (POWV) and Alkhurma hemorrhagic fever virus (AHFV) (family *Flaviviridae* genus *Flavivirus*); Severe fever with thrombocytopenia syndrome virus (SFTSV) and Heartland virus (HRTV) (family *Phenuiviridae* genus *Banyangvirus*); Colorado tick fever virus (CTFV) (family *Reoviridae*, genus *Coltivirus*), Bourbon virus (BRBV) (family *Orthomyxoviridae*, genus *Thogotovirus*) and African swine fever virus (ASFV) (family *Asfarviridae*, genus *Asfivirus*)^4,5^. Expanding activity zones and changing epidemiology have been documented for some of these viruses, such as for CCHFV around the Mediterranean Sea region, POWV in North America and AHFV in Africa^5^. In addition to these established tick-borne pathogens, a number of newly characterized viruses have been associated with human infections, including Jingmen Tick Virus (JMTV) and Alongshan virus (ALSV), (family *Flaviviridae*), Tacheng Tick Virus 1 (TTV1) and Songling virus (SGLV) (family *Nairoviridae*, genus *Orthonairovirus*), Tacheng Tick Virus 2 (TTV2) (family *Phenuiviridae* genus *Uukuvirus*) and Haseki Tick Virus (HTV) (family *Flaviviridae*)^6–11^. Although cases have been reported from China (JMTV, ALSV, TTV1, TTV2, SGLV), Kosovo (JMTV) and the Russian Federation (HTV), information on pathogenesis, distribution and public health impact of these viruses are currently scarce.

Expansion and spillover of tick-borne viruses may be easily overlooked until the emergence of symptomatic case clusters or local epidemics involving humans or domestic animals. Timely identification of the circulating pathogens and assessment of public health threats rely heavily on surveillance^2,4^. Metagenome-based screening, enabling analysis of the nucleic acid content of any sample without prior information facilitates bio- or xeno-surveillance, where ticks can be utilized as “blood bags” to screen pathogens over multiple hosts^12^. Metagenome-based screening by Nanopore Sequencing (NS) has been documented as a powerful tool to detect known and novel tick-borne viruses^13^. In this study, we analyzed pooled and subsequently individual ticks collected from several locations in Eastern Europe and the Black Sea region using NS and targeted amplification for several recently described tick-borne viruses.

## Results

In total, 1337 ticks, belonging to 11 species, were included in the study and screened in 217 pools. Virus sequences were detected in 101 pools (46.5%) and viruses documented as tick-borne human pathogens were observed in 16 (7.3%). In pools with detectable viruses, probable coinfections, evidenced by multiple virus sequences from various families/genera, were present in 31 (30.6%). Co-infections of human pathogens were not detected in pools. Tick species and virus detection prevalences according to the country of collection are provided in Table 1.

**Table 1:** Summary of tick species and virus detection.

Tick species	Poland		Bulgaria		Ukraine		Georgia	
	#	Pool	#	Pool	#	Pool	#	Pool
*Dermacentor marginatus*	–	–	3	1	–	–	16	9
*Dermacentor reticulatus*	66	13	0	0	718	43	65	23
*Haemaphysalis parva*	0	0	0	0	–	–	120	27
*Ixodes ricinus*	80	14	124	20	–	–	32	18
*Hyalomma marginatum*	0	0	2	1	–	–	6	6
*Haemaphysalis punctata*	–	–	17	2	–	–	12	10
*Haemaphysalis innermis*	–	–	19	3	–	–	29	12
*Haemaphysalis sulcata*	–	–	–	–	–	–	8	2
*Rhipicephalus bursa*	–	–	–	–	–	–	10	10
*Rhipicephalus turanicus*	–	–	8	1	–	–	1	1
*Rhipicephalus rossicus*	–	–	–	–	–	–	1	1
Total	146	27 (12.4%)	173	28 (12.9%)	718	43 (19.8%)	300	119 (54.8%)
Virus (+)		19 (70.3%)		17 (60.7%)		37 (86%)		28 (23.5%)
Pathogen (+)		5 (18.5%)		–		1 (2.3%)		10 (8.4%)

Viruses belonging to 21 separate taxa were documented in the tick pools (Table 2). Detected tick-borne human pathogens comprising TTV2, JMTV and TTV1 were present in 5.9% (n = 13), 0.9% (n = 2) and 0.4% (n = 1) of the pools, respectively. All three pathogens were present in ticks collected from Poland, whereas none could be identified in samples from Bulgaria. An association with tick species was observed for viruses, regardless of the country of collection. A notable exception was TTV2, which was detected mainly in *Dermacentor marginatus* and *Dermacentor reticulatus* ticks, and also in a *Haemaphysalis punctata* pool from Georgia. *Ixodes ricinus* harbored the broadest virus diversity (10 virus taxa) and all JMTV detections. Information on individual pools with detectable virus sequences are provided in Supplementary Tables S1 and S2.

**Table 2:** Virus detection prevalences in pooled ticks according to country of collection and species.

Virus	Location				Tick species					
	Poland (n = 27, 12.4%)	Bulgaria (n = 28, 12.9%)	Ukraine (n = 43, 19.8%)	Georgia (n = 119, 54.8%)	*I. ricinus* (n = 52, 23.9%)	*D. reticulatus* (n = 79, 36.4%)	*D. marginatus* (n = 10, 4.6%)	*H. marginatum* (n = 7, 3.2%)	*Rhipicephalus spp.* (n = 13, 5.9%)	*Haemaphysalis spp.* (n = 56, 25.8%)
Tacheng tick virus 2 (n = 13; 5.9%)	3 (11.1%)	–	1 (2.3%)	9 (7.5%)	–	9 (11.3%)	3 (30%)	–	–	1 (1.8%)
Tacheng tick virus 1 (n = 1, 0.4%)	1 (3.7%)	–	–	–	–	1 (1.9%)	–	–	–	–
Jingmen tick virus (n = 2, 0.9%)	1 (3.7%)	–	–	1 (0.8%)	2 (3.8%)	–	–	–	–	–
Changping tick virus 1 (n = 25, 11.5%)	–	–	19 (44.1%)	6 (5%)	–	25 (31.6%)	–	–	–	–
Norwavirus (n = 22, 10.1%)	7 (25.9%)	15 (53.5%)	–	–	22 (42.3%)	–	–	–	–	–
*D. reticulatus* pestivirus-like virus 1 (n = 14, 6.4%)	9 (33.3%)	–	–	5 (4.2%)	–	14 (17.7%)	–	–	–	–
*D. reticulatus* uukuvirus (n = 9, 4.1%)	5 (18.5%)	–	–	4 (3.3%)	–	9 (11.3%)	–	–	–	–
Bole tick virus 4 (n = 4, 1.8%)	–	1	–	3 (2.5%)	–	–	–	4 (57.1%)	–	–
Bole tick virus 3 (n = 3, 1.3%)	–	–	–	–	–	–	–	–	3 (23.1%)	–
Iflavirus (n = 3, 1.3%)	1 (3.7%)	2 (7.1%)	–	–	3 (5.7%)	–	–	–	–	–
Tick phlebovirus (n = 3, 1.3%)	–	1 (3.5%)	–	2 (1.6%)	–	–	–	–	2 (15.3%)	1 (1.8%)
Hubei toti-like virus 24 (n = 2, 0.9%)	–	–	–	2 (1.6%)	–	1 (1.9%)	2 (20%)	–	–	–
*I.scapularis* bunyavirus *(n* = *2, 0.9%)*	–	2 (7.1%)	–	–	2 (3.8%)	–	–	–	–	–
Norway luteo-like virus 3 (n = 2, 0.9%)	2 (7.4%)	–	–	–	2 (3.8%)	–	–	–	–	–
Norway partiti-like virus 1 (n = 2, 0.9%)	–	–	–	2 (1.6%)	2 (3.8%)	–	–	–	–	–
Bole tick virus 2 (n = 1, 0.4%)	–	–	–	1 (0.8%)	–	–	–	1 (14.2%)	–	–
Chimay rhabdovirus (n = 1, 0.4%)	–	1 (3.5%)	–	–	1 (1.9%)	–	–	–	–	–
Norway mononegavirus 1 (n = 1, 0.4%)	1 (3.7%)	–	–	–	1 (1.9%)	–	–	–	–	–
Serbia narna-like virus (n = 1, 0.4%)	–	1 (3.5%)	–	–	1 (1.9%)	–	–	–	–	–
Taishun tick virus (n = 1, 0.4%)	–	–	–	1 (0.8%)	–	–	–	1 (14.2%)	–	–
Uukuniemi virus (n = 1, 0.4%)	–	–	–	1 (0.8%)	1 (1.9%)	–	–	–	–	–

### Individual PCR and NS screening

We performed targeted PCR and additional NS on individual ticks from pools with detectable viral pathogens. Fifty-nine individual samples from 15 pools were amplified and subsequently sequenced, including samples with expected band sizes for TTV2 and JMTV observed in electrophoresis (n = 31), selected samples negative in targeted amplification (n = 6) and controls comprising a female and a male *I. ricinus* specimens (n = 2, 39 in total). A pool with TTV2 detection from Ukraine and JMTV detection from Poland could not be included in PCR screening due to the lack of sufficient material for testing.

### TTV2 findings

Using targeted PCR, we screened all individuals from 10 pools from Georgia and 3 pools from Poland, which were NS positive for TTV2 (n = 12) and closely-related *Dermacentor reticulatus* uukuvirus (DRUV) (n = 1). Subsequently, we sequenced all PCR positive (n = 29) and selected PCR negative (n = 3) individuals. Expected viruses, based on NS sequencing, were observed in 19 individual samples—18 PCR (+) and 1 PCR (−). The largest contigs were characterized as TTV2 (n = 15) and DRUV (n = 4) by BLAST, with mapped read counts of 3–86 and sizes between 332 and 5659 bp on the L and S genome segments. Breakup of pooled and single samples and virus detections are provided in Supplementary Table S2.

**Figure 1:**
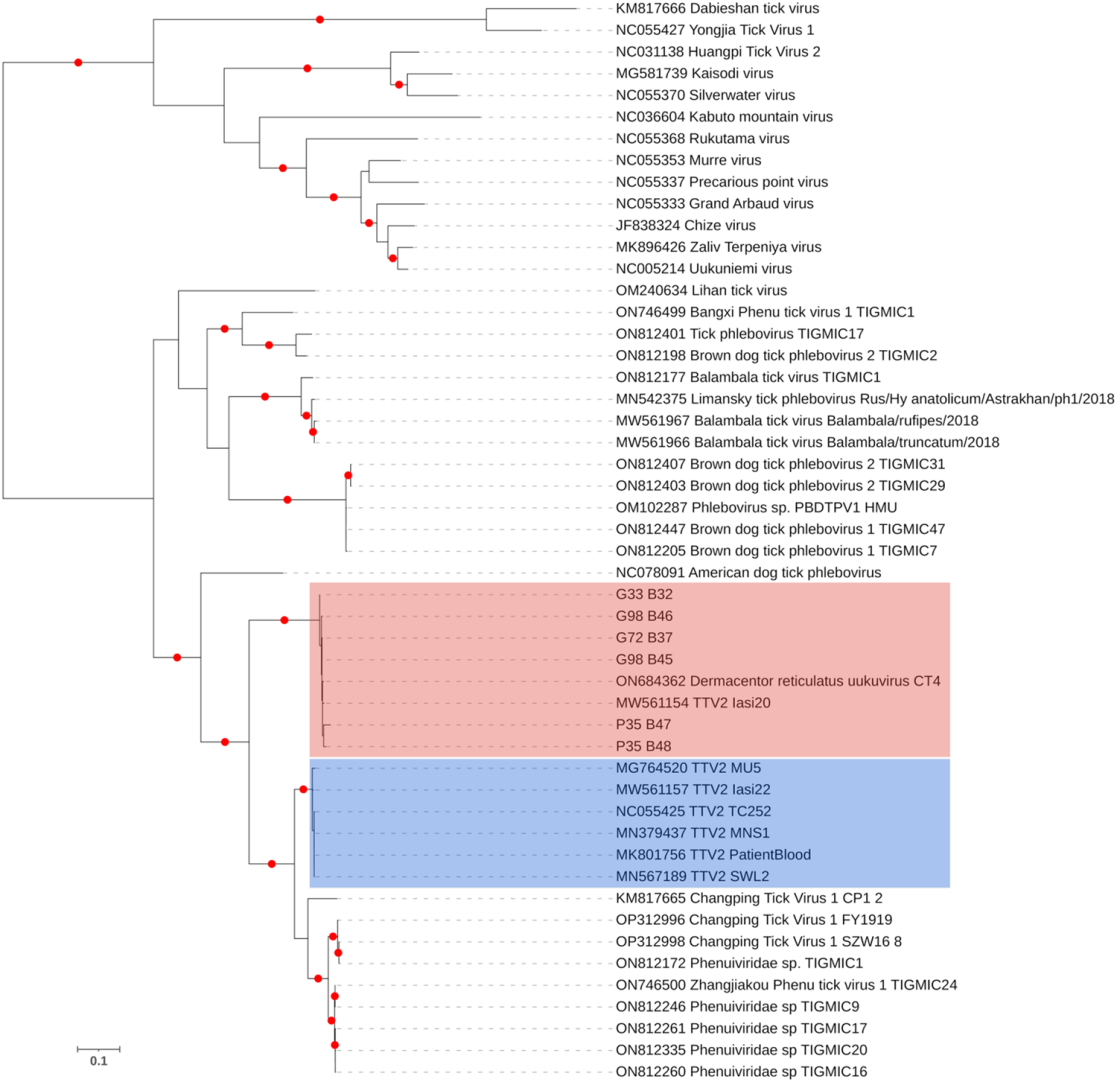
The maximum likelihood consensus tree of the phenuivirus polymerase sequences (783 amino acids), constructed using 1000 replicates. Branches achieving ≥ 95% bootstrap support are annotated with red dots. Tacheng Tick Virus 2 (TTV2) lineages are marked. Viruses are indicated by GenBank accession, name and isolate identifier where available. Sequences detected in the study are indicated by sample IDs.

We further analyzed 6 larger TTV2 L segment contigs (> 2330 bp) from different individuals and collection sites. Pairwise comparisons revealed up to 9.9% and 2.5% diversity on nucleotide and amino acid alignments, respectively (Supplementary Table S3). In the phylogenetic tree, two well-demarcated TTV2 clusters were observed, where the sequences identified in this study clustered with a TTV2 and DRUV sequence from Eastern Europe, distinct from other TTV2s from Europe and Asia (Fig. 1).

A large portion (1885 of 2189 amino acids) of the TTV2 L segment, encoding for the RNA-dependent RNA polymerase (viral replicase), was available in two individual samples from Georgia (G33-B32, G98-B45, GenBank accessions). The sequences demonstrated 6.6% and 1.7% divergence based on nucleotide and putative amino acid alignments, respectively, which increased to 32.1% nucleotide and 5.7% amino acid divergence in pairwise comparisons with the closest relative (TTV2 strain Iasi20) (Supplementary Table S3). Conserved domain searches revealed the N-terminal endonuclease motif (PFAM15518) required for cap-dependent transcription (amino acids 1–45), Bunyavirus RNA-dependent RNA polymerase (PFAM04196) (amino acids 529–1223) and associated domain (PFAM12603) (amino acids 101–344) in the putative viral proteins. Phylogeny construction based on these sequences confirmed the findings from shorter TTV2 contigs and displayed a separate grouping of these sequences and DRUV (Supplementary Fig. S1). No strong signal for potential recombination was noted among TTV2/DRUV sequences.

### TTV1 findings

**Figure 2:**
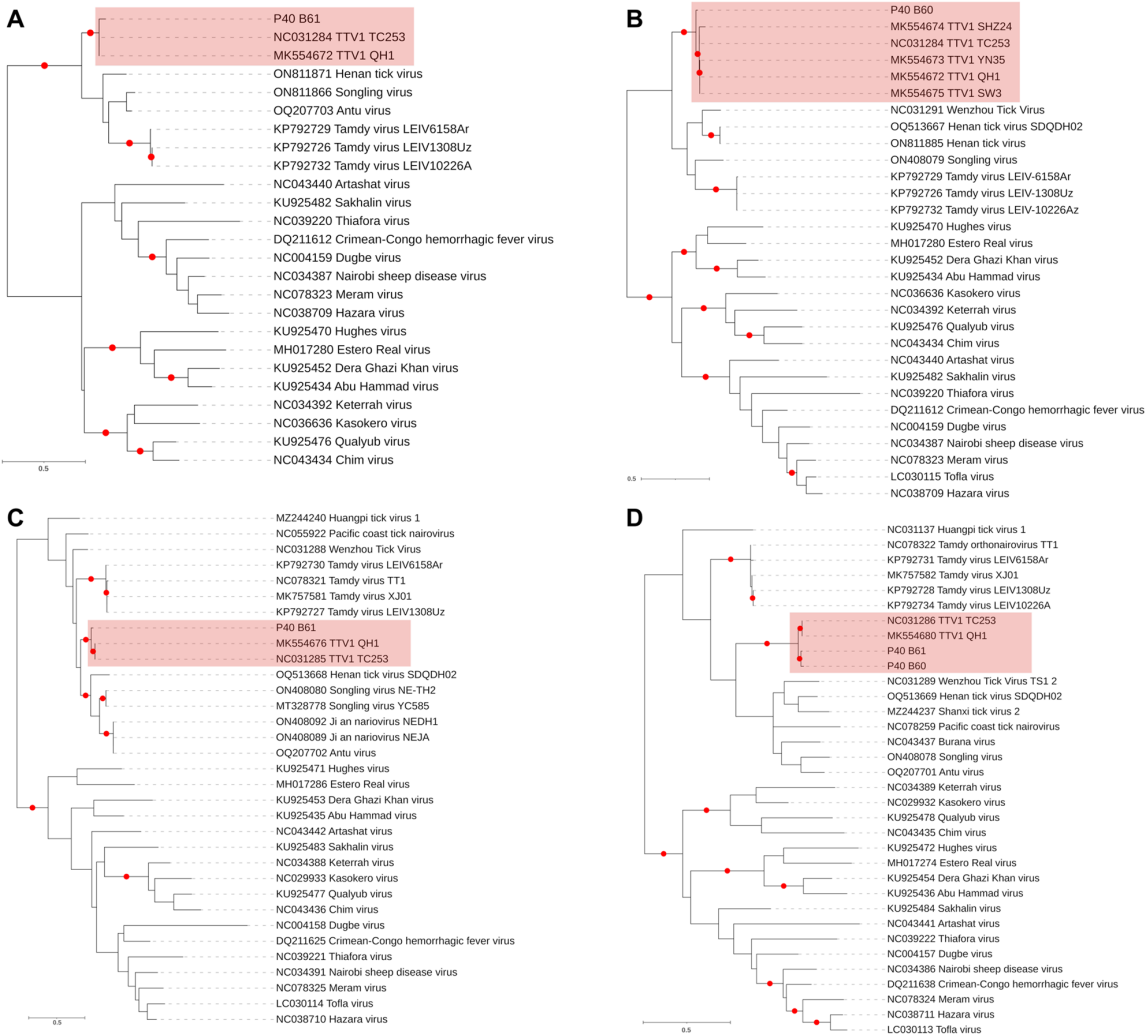
The maximum likelihood consensus tree of the nairovirus polymerase (**A**: 335 amino acids, **B**: 359 amino acids), glycoprotein precursor (**C**: 115 amino acids) and nucleocapsid (D: 135 amino acids) sequences. The trees are constructed using 1000 replicates. Branches achieving ≥ 95% bootstrap support are annotated with red dots. Tacheng Tick Virus 1 (TTV1) sequences in each alignment are marked. Viruses are indicated by GenBank accession, name and isolate identifier where available. Sequences detected in the study are indicated by sample IDs.

Targeted PCR was carried out in individual *D. reticulatus* samples from Poland, with TTV1 sequences identified as a single pool (Table 2). Despite all samples being negative in PCR, we selected three samples and performed individuals NS. Interestingly, TTV1 could be detected in two of these individuals with contig sizes of 391–997 bp, representing all three virus genome segments (L, M and S) in one sample and two segments (L and S) in another. Pairwise sequence comparisons with available TTV1 genomes showed 19.4% and 7.3% divergence based on nucleotide and amino acid alignments, respectively (Supplementary Table S3). The partial S segment contigs identified in both samples did not cover the primer binding sites utilized for the screening PCR. We further carried out maximum likelihood phylogenies for each virus genome segment (performed individually for the L segment due to non-aligning contigs). In all trees, TTV1 sequences from Poland grouped with previously reported TTV1 isolates, distinct from other orthonairoviruses (Fig. 2).

### JMTV findings

JMTV sequences were detected by NS in two *I. ricinus* pools from Poland and Georgia (Table 2). In these samples, only virus replicase sequences (located on genome segment 1) with low abundance (2–3 mapped reads) were noted. We could not perform targeted JMTV screening in individual ticks from Georgia, due to the insufficient material available. Two out of 9 individual samples tested were observed as positive. However, subsequent NS performed on both positive samples revealed a single read of 285 bp in one sample, with a BLAST similarity of 90.97% to a JMTV replicase sequence observed as integrated into the *I. ricinus* chromosome (Supplementary Table S2).

### Other viruses

In addition to documented pathogens described above, 18 viral taxa were detected in pooled ticks (Table 2). Changping tick virus 1 (family *Phenuiviridae*, genus *Phlebovirus*) was detected in 11.5% of the pools, which were exclusively *D. reticulatus* ticks. Sequences with highest identities to members of the *Norwavirus* genus (family *Nairoviridae*) were also common, detected in 10.1% of the pools and in *I. ricinus* ticks. Our phylogenetic analyses based on Bunyavirus polymerase and nucleocapsid amino acid alignments revealed diverse virus sequences related to Grotenhout virus and other proposed members, distinct from the recently described congeneric pathogen Beiji nairovirus (Supplementary Fig. S2)^7,14,15^.

Another frequently observed virus was *D. reticulatus* Pestivirus-like Virus 1 (DRPV1) (family *Flaviviridae*, genus *Pestivirus*), present in 6.4% (14/217) of pooled and 23.1% (9/39) of single *D. reticulatus* ticks. This virus is related to HTV (unclassified *Riboviria*), with 91.8% and 94.4% nucleotide and putative amino acid identities, respectively. Despite being absent in pooled ticks, sequences with highest BLAST identities to HTV were observed in four individual *D. reticulatus* samples from Georgia and Poland, with a prevalence of 10.2% (4/39) (Supplementary Table S2). Alignment and phylogenetic analysis revealed subgrouping of the sequences with particular HTV isolates as well as DRPV1. Further phylogeny construction using contigs of various sizes from pooled samples also confirmed a close relationship between these viruses, probably representing geographically segregated lineages (Supplementary Fig. S3).

Virus taxa observed in pooled NS were also noted in individual ticks. However, additional viral taxa (n = 4) including Tacheng Tick Virus 5 (TTV5; unclassified *Riboviria*), Tacheng Tick Virus 3 (TTV3; *Rhabdoviridae*), *Dermacentor reticulatus* Rhabdovirus 1 (DRR1; *Rhabdoviridae*) and *Betaricinrhavirus chimay* (*Rhabdoviridae*) were identified during analyses of individual ticks, with < 10 mapped reads (Supplementary Table S2).

## Discussion

Our pooled and individual metagenome-based tick screening using NS revealed 25 virus taxa including four tick-borne pathogens in samples collected from countries from Eastern Europe and around the Black Sea. Known tick-borne viruses endemic in the region, such as CCHFV or TBEV, were not detected, owing to the low prevalence of these viruses within the tick populations in targeted sites or sampling bias. However, we identified recently described tick-borne pathogens TTV2, TTV1, JMTV (7.2% in pools) and HTV (10.2% in individuals) in the sample cohort and produced abundant virus genome data for further downstream analysis.

Among the tick-borne viral pathogens identified, TTV2 was the most frequent, detected in 5.9% of the tick pools (13/217) from several sites in three of the four countries of collection, including Eastern Europe (Poland and Ukraine) and the Asian Black Sea region (Georgia). Detections were further confirmed in individual targeted PCR and NS (Supplementary Table S2). Classified in the *Uukuvirus tachengense* species, TTV2 was initially described during a metagenomic investigation of arthropods from China and has since been detected in *D. marginatus*, *Dermacentor nuttalli*, *Dermacentor silvarum*, and *Hyalomma asiaticum* ticks^9,16,17^. Subsequently, it has been documented as the causative agent in a patient with tick-associated febrile disease from China, with implications of person-to-person transmission through droplets or direct contact with body fluids^9^. Further detections of TTV2 in *Hyalomma* / *Dermacentor* spp. from Kazakhstan (according to GenBank records) and *D. reticulatus* from Romania are reported^18^. TTV2 is also documented in *D. marginatus* and the prominent CCHFV vector, *H. marginatum*, from ecologically diverse locations in Asia Minor, with evidence for trans-stadial or horizontal virus transmission among ticks^19,20^. Interestingly, we observed two distinct TTV2 clades in our phylogenetic analysis, where one of the groups also included DRUV, an unclassified *Ribovirus* separately detected in 4.1% of the tick pools in the study. DRUV was recently described from Croatia in *D. reticulatus* pools^21^, but to date there is scant information. Genomes of both TTV2 and DRUV consist of L and S segments, while seemingly lacking the glycoprotein encoding M segment, even in cell culture grown isolates^9,21^. The presence of TTV2 and DRUV divergent clades and human exposure in Europe or around the Black Sea region require further investigation.

We further detected TTV1 in 0.4% of the pools, comprising *D. reticulatus* from Poland. Although no amplification was observed in virus specific targeted PCR, individual NS revealed several contigs of varying sizes, encompassing all virus genome segments. Subsequent phylogenetic analyses placed all contigs with the global TTV1 sequences as a separate group within the *orthonairovirus* genus (Fig. 2). This is the first documentation of TTV1 in Europe. Currently, TTV1 is classified as the sole taxon in the *Tacheng orthonairovirus* species (genus *Orthonairovirus*, family *Nairoviridae*)^15^. Initially described during virus discovery efforts in arthropods from China, TTV1 was also detected in febrile diseases associated with tick bites, and as a co-infecting agent in a case with Rickettsial fever and meningitis^8,16,22^. Virus excretion patterns in infected individuals suggested possible transmission by direct contact with body fluids or droplets, similar to CCHFV, as noted for TTV2^8,9^. Virus exposure was further documented in local sheep, cattle and human populations in China, with *Dermacentor* spp. suggested as probable vectors^9^. Virus genomes were detected in *D. marginatus*, *D. silvarum*, *D. nuttalli*, and *H. asiaticum* ticks in China, and also identified from spleen tissues of great gerbils (*Rhombomys opimus*)^9,23^. Recently, the virus has been documented in pools of *Hyalomma aegyptium* from southern Turkey^24^. It remains to be determined whether the lack of detection in targeted amplification in our tick cohort reflects genome diversity or low sensitivity of the PCR.

JMTV is the best studied emerging tick-borne pathogen observed in our cohort. It was identified in 0.9% of the samples, comprising *I. ricinus* samples collected from Poland and Georgia (Table 2). Classified within the *Flaviviridae* family, JMTV and related viruses (Jingmenviruses) appear to be widely distributed, having been identified in a diverse spectrum of tick species as well as in bats, cattle and rodents from Asia, Africa, Europe and the Americas^25^. JMTV and Alongshan virus (another Jingmenvirus) have been documented to cause human infections and subsequent seroconversion, with further evidence for exposure in domestic animals and humans^6,7^. Co-infections with JMTV were reported among individuals with CCHFV in Kosovo, with possible impacts on disease outcome^26^. In Europe, JMTV-infected ticks were reported from Romania, Serbia and the Thrace region of Turkey, while Alonghsan virus detection is more pronounced in northern Europe^25–28^. Recently, the integration of the JMTV polymerase gene into *I. ricinus* genome was documented, in ticks of both sexes and *I. ricinus* embryo-derived tick cell line IRE/CTVM19^29^. The integrated fragment of the viral genome was reported to be variable in size and forming several genovariants, with an average of 1.5 copies per cell. Several tick species other than *I. ricinus* were further suggested to contain the integrated segments. In field collected *I. ricinus* samples, the integration event was observed with a prevalence of up to 34.3% in various regions of Russia^29^. The JMTV findings in our study strongly suggest integrated virus sequences being detected, rather than replicating viruses. We also have previously reported NS as capable of detecting all JMTV genome segments, surpassing the sensitivity of screening by nested PCR^13^. Documentation of virus integration in tick vectors also has ramifications for PCR-based screening, which may require additional genome targets or steps to exclude amplification of integrated fragments.

Our findings on HTV and DRPV1 demand further discussion. We detected DRPV1 in 6.4% and 23.1% of the pooled and individual *D. reticulatus* samples, respectively. This virus was first described in *D. reticulatus* from Croatia, sharing significant NS3–NS5 protein homologies with members of the *Pestivirus* genus^21^. It is closely related to other recently documented pestivirus-like viruses, Bole Tick Virus 4 (BTV4), Trinbago Virus (TBOV), as well as HTV^21^. HTV genomes were recently documented in retrospectively screened sera from individuals with febrile disease associated with tick bites, with prevalences of 0.9–9.3% in different regions of the Russian Federation, with and without TBEV or *Borrelia* spp.^11^. We detected HTV in 10.2% of the single *D. reticulatus* samples from Georgia and Poland and observed clustering of the sequences with HTVs as well as DRPV1 in phylogenetic analyses. Given these findings and limited sequence variation between complete HTV and DRPV1 polyprotein coding sequences, these viruses represent sub-lineages of the same taxon, observed in geographically segregated regions. The capacity of either virus to produce human symptomatic infections requires further investigation.

Finally, we detected Norwaviruses, distinct from Beiji nairovirus, in 10.1% of the pools, comprising *I. ricinus* ticks from Bulgaria and Poland. *Norwavirus* is a recently described genus in the family *Nairoviridae*, which currently includes Grotenhout Virus (GRHV) as the sole member of the *Grotenhout norwavirus* species^15^. Closely related viruses have been documented in northern Europe and central Asia, such as Pustyn Virus (PTV) and Norway Nairovirus 1 (NWNV1), but these strains currently remain taxonomically unclassified^7,14,15^. Similar to TTV2, norwaviruses lack the genomic segment M, which encodes for nairovirus glycoproteins. Beiji nairovirus (BJNV), a norwavirus-related virus, was documented as a potential agent causing tick-borne febrile diseases in China, supported by experimental inoculations^7^. The public health implications of norwaviruses currently remain underexplored.

In conclusion, our findings reveal several tick-borne viral pathogens, recently reported from various regions from Asia, to be present in various locations in Eastern Europe and Asian Black Sea region. In any of the sampling regions, actual prevalences of individual viruses is hard to assess, due to our cross-sectional collection and non-targeted screening strategy employed in the study. Moreover, local virus clades or integrated virus genomes might undermine targeted screening due to sequence variation (TTV1) or amplification of the integrated fragments (JMTV). Currently, the lack of standardized antibody testing further hinders investigation of previous exposures and serological diagnosis. Nevertheless, these viruses should be considered in the diagnostic assessment of symptomatic cases associated with tick bites and tested when possible. NS proves to be a useful tool for monitoring tick-associated pathogens in pooled and individual samples.

## Methods

### Sample collection, processing and barcoding

**Figure 3:**
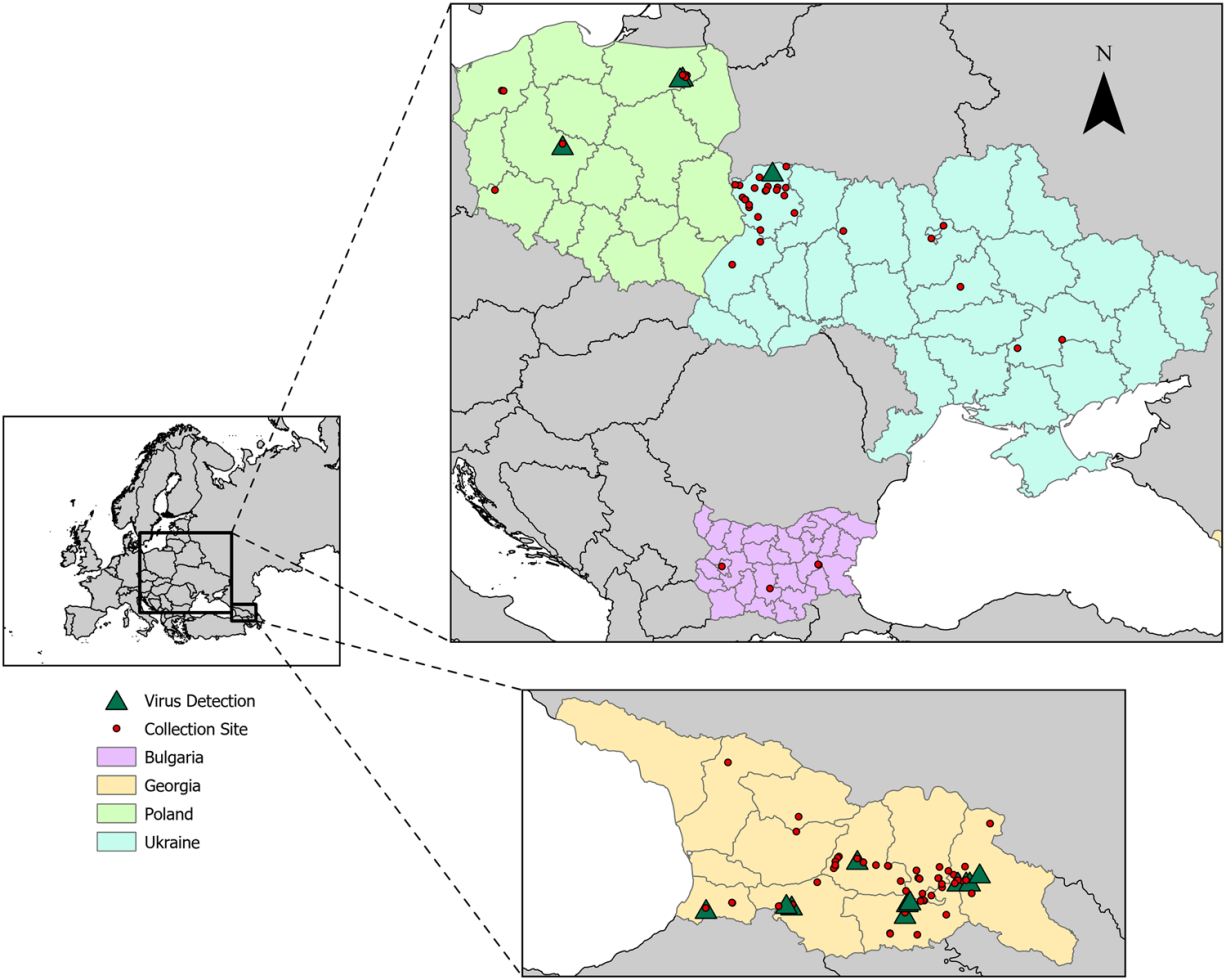
Map indicating the tick sampling locations in the study. Sites with detectable human pathogens are marked by a triangle.

Questing adult ticks were collected via drag/flagging at 75 ad hoc sites in Bulgaria (2021–2022), Poland (2021), Georgia (2021) and Ukraine (2012–2013) (Fig. 3). The samples were morphologically identified using appropriate keys^30^ and stored at − 80 °C. Subsequently, samples from Bulgaria, Poland and Georgia were shipped to Walter Reed Biosystematics Unit (WRBU), Museum Support Center, Smithsonian Institution, MD, USA, in dry ice. Samples from Ukraine were pooled according to species and collection site, processed locally and nucleic acids were shipped to WRBU for further processing. The dorsal and ventral sides of intact specimens were imaged using IDX machine (Vectech, Baltimore, MD, USA).

Nucleic acid extraction was carried out individually following homogenization in sterile 1 × PBS (ThermoFisher Scientific, Waltham, MA, USA) and ATL Lysis Buffer with regent DX (Qiagen, Valencia, CA, USA) with 5 mm stainless steel grinding balls (OPS Diagnostics, Lebanon, NJ, USA) using TissueLyser (Qiagen, Valencia, CA, USA). Following centrifugation, the supernatant was purified using the IndiMag Pathogen Kit (Indical Bioscience, Leipzig, Germany) on BioSprint 96 DNA/RNA Purification System (Qiagen, Valencia, CA, USA), according to the manufacturer recommendations.

Morphological identification of the individual ticks was confirmed using DNA barcoding, by amplification of the 658 bp region of the mitochondrial cytochrome c oxidase subunit I (COI) gene, as described previously^31,32^. Subsequently, 5 µl of the individual tick nucleic acids were pooled according to species, sex and locality of collection (up to a maximum of 9), and stored in − 80 °C.

### Nanopore sequencing (NS)

Pooled nucleic acids were subjected to cDNA synthesis using NEBNext Ultra II RNA First Strand and Non-Directional RNA Second Strand Synthesis modules, utilizing a random primer mix (New England Biolabs, Ipswich, MA, USA). Double stranded cDNA was cleaned up using Agencourt AMPure XP reagent (Beckman Coulter Biosciences, Indianapolis, IN, USA) and quantitated by Qubit dsDNA HS Assay Kit (ThermoFisher Scientific, Waltham, MA, USA). The NEBNext Ultra II End repair/dA-tailing and Quick Ligation modules (New England Biolabs, Ipswich, MA, USA), and Ligation Sequencing Kit SQK-LSK109 (Oxford Nanopore Technologies, Oxford, UK) were used to prepare libraries, which were further quantitated by Qubit (ThermoFisher Scientific, Waltham, MA, USA) using 1 × dsDNA HS Assay Kit. Each sample was separately barcoded with the Native Barcoding Expansion 96-EXP-NBD 196 (Oxford Nanopore Technologies, Oxford, UK). An epMotion 5075 workstation (Eppendorf, Hamburg, Germany) was used for automated liquid handling and library preparation, carried out according to the manufacturer recommendations. Sequencing libraries containing 20–24 barcoded pools were loaded on a single ONT R9.4.1 flow cell and run on the GridION sequencing device (Oxford Nanopore Technologies, Oxford, UK) for 48 h.

### Targeted viral pathogen detection

Nested polymerase chain reaction (PCR) was used to amplify JMTV, TTV1 and TTV2 in single tick samples with the corresponding virus sequences observed in pooled NS. We employed previously published primer sets and conditions for each assay, in a 30 µl total volume per sample. The assays targeted JMTV NS5-like protein on segment 1 and the nucleocapsid protein on segment S for TTV1 and TTV2^8,9,33^. The amplicons of the nested amplification (JMTV: 394 bp, TTV1: 328 bp, TTV2: 252 bp) were visualized by electrophoresis on 1.5% agarose gels using GelGreen Nucleic Acid Gel Stain (Biotium Inc., Fremont, CA, USA).

### Data analysis

Following NS, base-calling and demultiplexing was carried out on the GridION with the MinKNOW operating software v21.11.7 (Oxford Nanopore Technologies) and Guppy v5.1.13^34^. Raw reads were trimmed with Porechop to remove adapter sequences and then filtered with NanoFilt to remove reads with q-scores ≤ 9 and read lengths ≤ 100 bp^34,35^. Tick genome data were then removed as hosts using Minimap2 v2.24 and Samtools v1.9^36,37^. Subsequently, the data was aligned to the National Center for Biotechnology Information (NCBI) non-redundant (NR) database using DIAMOND v2.0.14^38^ and visualized using MEGAN6 (v6.23.2)^39^.

Sequences were handled using Geneious Prime (v2022.2.1) (Biomatters Ltd., Auckland, New Zealand). BLASTn and BLASTp algorithms were used for similarity searches in the NCBI database^40^. Read mapping was carried out using Minimap2 plug-in for Geneious Prime, with default settings optimized for nanopore data. Longest reads were used for similarity assessment when contigs were not available. Alignment and pairwise sequence comparisons were carried out using CLUSTAL W^41^. Potential genetic exchange and recombination were assessed using the RDP4 software in the default settings^42^. Protein domain and motif searches were performed using the NCBI conserved domain search tool and MOTIF Search in the PFAM database^43,44^. Phylogenetic analysis was performed on sequences using IQ-TREE 2^45^. Optimal evolutionary models and partitioning schemes were determined for nucleotide and amino acid sequence alignment using the automatic model selection tools (-m MFP + MERGE). The maximum likelihood extended majority-rule consensus trees were constructed using the ultrafast bootstrap approximation approach (UFBoot) for 1000 replicates^46^.

### Supplementary Information

Supplementary Figure 1.

Supplementary Figure 2.

Supplementary Figure 3.

Supplementary Tables.

## Data Availability

Consensus DNA sequences, specimen images, and edited chromatograms are available on BOLD under project “SHTRP and SHTRB”. All data generated in this study are available in the NCBI Biosample and Sequence Read Archive under the BioProject PRJNA878809 (SRR21504630-SRR21504591) and GenBank with the accession numbers OP432043–OP432053.
