# Supplementary material for: The expanding range of emerging tick-borne viruses in Eastern Europe and the Black Sea Region
Source: Sci Rep. 2023 Nov 14;13:19824. doi: 10.1038/s41598-023-46879-2 (PMC10646066; doi:10.1038/s41598-023-46879-2)

Supplementary Figure S1. The maximum likelihood consensus tree of the phenuivirus polymerase sequences (1,931 amino acids), constructed using 1,000 replicates. Branches achieving ≥95% bootstrap support are annotated with red dots. Viruses are indicated by GenBank accession, name and isolate identifier where available. Sequences detected in the study are indicated by sample IDs.

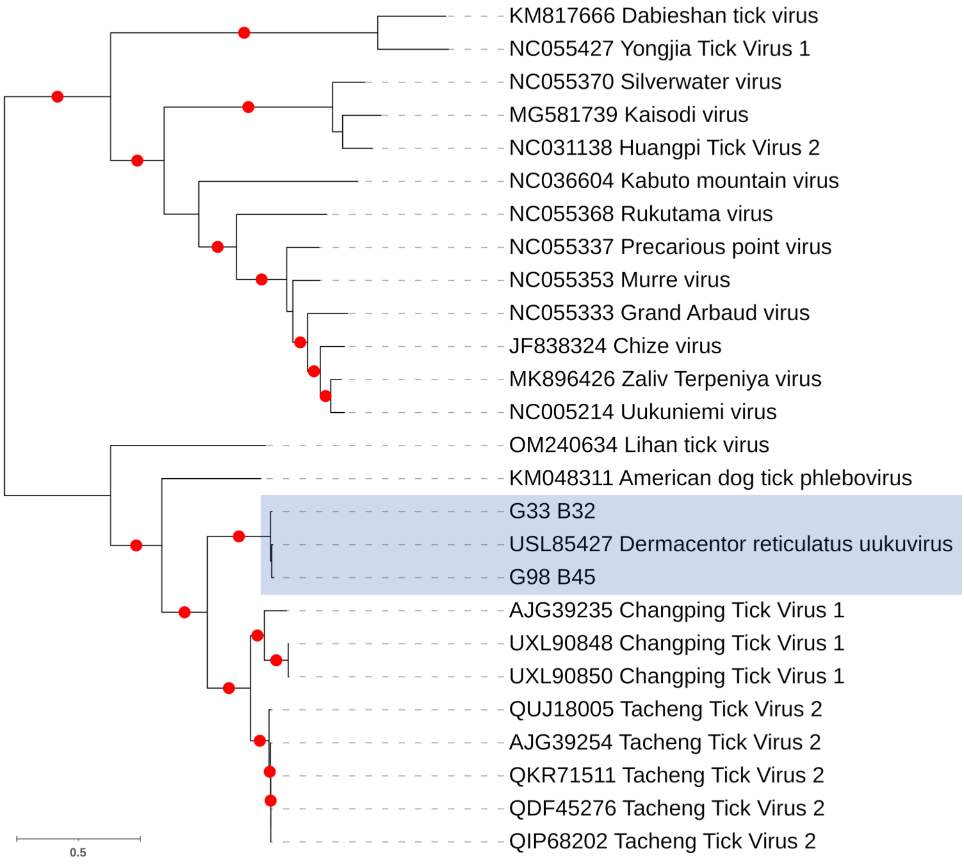

Supplement: Supplementary file 1 — Supplementary Figure 1. [file 41598_2023_46879_MOESM1_ESM.pdf]
