# Supplementary material for: The expanding range of emerging tick-borne viruses in Eastern Europe and the Black Sea Region
Source: Sci Rep. 2023 Nov 14;13:19824. doi: 10.1038/s41598-023-46879-2 (PMC10646066; doi:10.1038/s41598-023-46879-2)

Supplementary Figure S2. The maximum likelihood consensus tree of the norwavirus and nairovirus polymerase (A: 2,588 amino acids) and nucleocapsid (B: 526 amino acids) sequences. The trees are constructed using 1,000 replicates. Branches achieving ≥95% bootstrap support are annotated with red dots. Norwaviruses other than Beiji nairovirus are marked. Viruses are indicated by GenBank accession, name and isolate identifier where available. Sequences detected in the study are indicated by sample ID and country of detection.

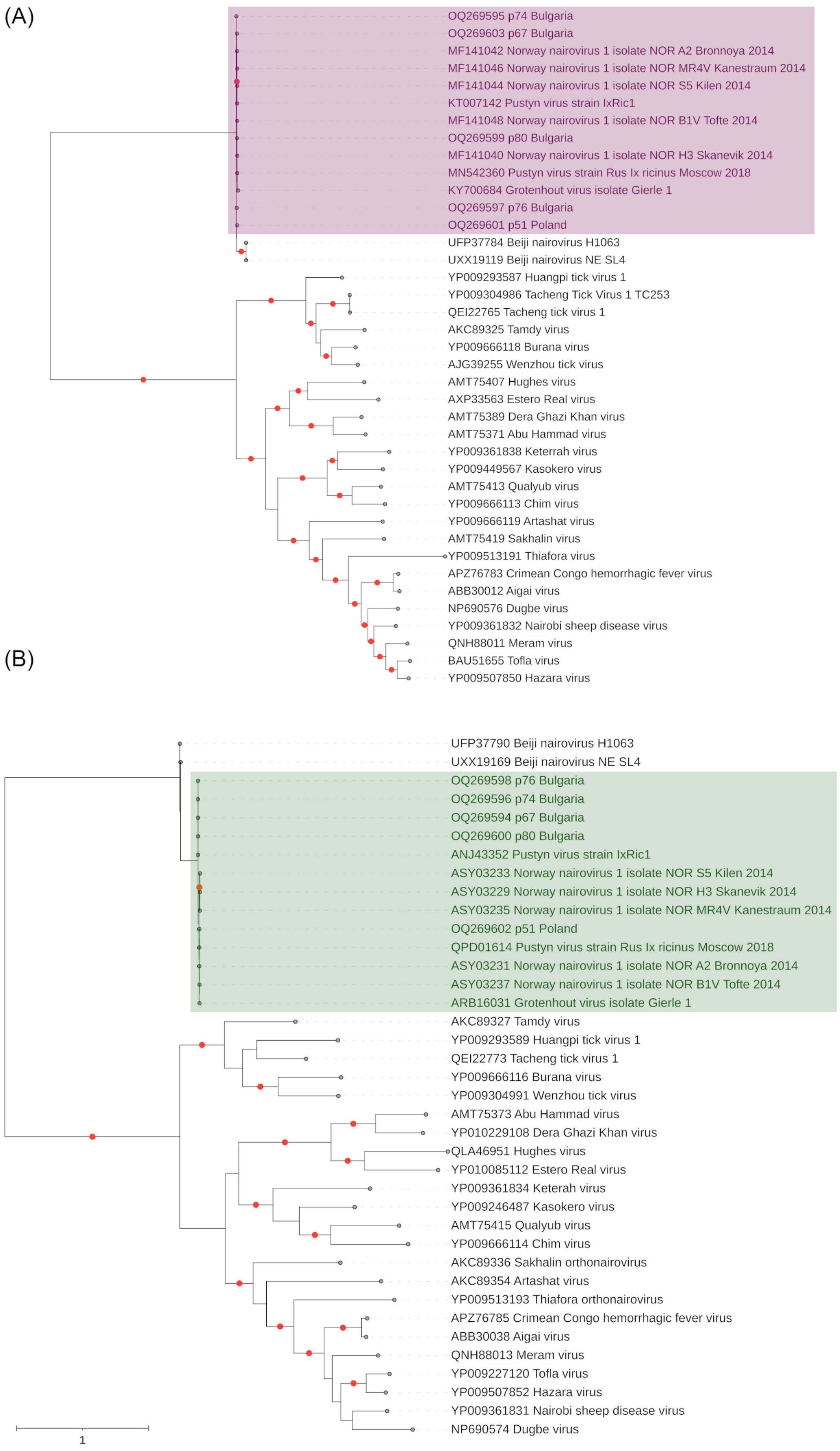

Supplement: Supplementary file 2 — Supplementary Figure 2. [file 41598_2023_46879_MOESM2_ESM.pdf]
