# Supplementary material for: The expanding range of emerging tick-borne viruses in Eastern Europe and the Black Sea Region
Source: Sci Rep. 2023 Nov 14;13:19824. doi: 10.1038/s41598-023-46879-2 (PMC10646066; doi:10.1038/s41598-023-46879-2)

**Supplementary Figure S3.** The maximum likelihood consensus tree of the flavivirus polyprotein (A: 118 amino acids, B: 454 amino acids) sequences. The trees are constructed using 1,000 replicates. Branches achieving ≥95% bootstrap support are annotated with red dots. Viruses are indicated by GenBank accession, name and isolate identifier where available. Haseki Tick Virus and related viruses are marked. Sequences detected in the study are indicated by sample IDs.

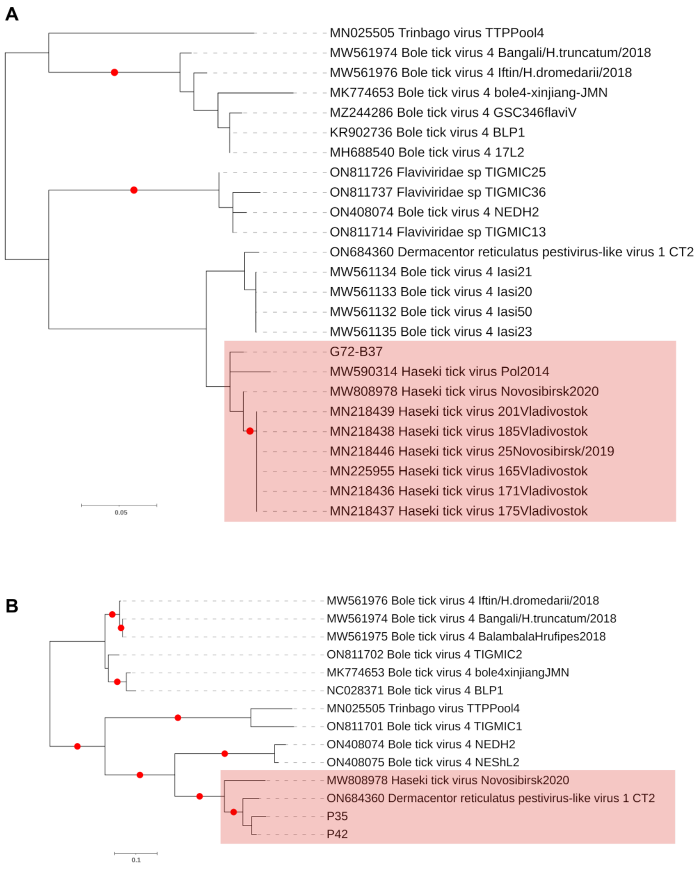

Supplement: Supplementary file 3 — Supplementary Figure 3. [file 41598_2023_46879_MOESM3_ESM.pdf]
